# A screen for over-secretion of proteins by yeast based on a dual component cellular phosphatase and immuno-chromogenic stain for exported bacterial alkaline phosphatase reporter

**DOI:** 10.1186/1475-2859-12-36

**Published:** 2013-04-19

**Authors:** Ferez S Nallaseth, Stephen Anderson

**Affiliations:** 1 Department for Molecular Biology and Biochemistry, Center for Advanced Biotechnology and Medicine Rutgers, The State University of New Jersey, 679 Hoes Lane West, Piscataway, NJ 08854, USA

**Keywords:** Cellular phosphatase reference activity, Dual component chromogenic and immuno-chromogenic stains, *P. pastoris*, *AOX1*, α-sec-EAP (α-sec*MAT*, *E.coli* Alkaline Phosphatase), Protein export, Chemical mutagenesis, Over-secretor mutants

## Abstract

**Background:**

To isolate over-secretors, we subjected to saturation mutagenesis, a strain of *P.pastoris* exporting *E. coli* alkaline phosphatase (EAP) fused to the secretory domain of the yeast α factor pheromone through cellular *PHO1/KEX2* secretory processing signals as the α-sec-EAP reporter protein. Direct chromogenic staining for α-sec-EAP activity is non-specific as its NBT/BCIP substrate cross-reacts with cellular phosphatases which can be inhibited with Levulinic acid. However, the parental E(P) strain only exports detectable levels of α-sec-EAP at 69 hours and not within the 36 hour period post-seeding required for effective screening with the consequent absence of a reference for secretion. We substituted the endogenous cellular phosphatase activity as a comparative reference for secretion rate and levels as well as for colony alignment while elevating specificity and sensitivity of detection of the exported protein with other innovative modifications of the immuno-chromogenic staining application for screening protein export mutants.

**Results:**

Raising the specificity and utility of staining for α-sec-EAP activity required 5 modifications including some to published methods. These included, exploitation of endogenous phosphatase activity, reduction of the cell/protein burden, establishment of the direct relation between concentrations of transcriptional inducer and exported membrane immobilized protein and concentrations of protein exported into growth media, amplification of immuno-specificity and sensitivity of detection of α-sec-EAP reporter enzyme signal and restriction of staining to optimal concentrations of antisera and time periods. The resultant immuno-chromogenic screen allows for the detection of early secretion and as little as 1.3 fold over-secretion of α-sec-EAP reporter protein by E(M) mutants in the presence of 10 fold -216 fold higher concentrations of HSA.

**Conclusions:**

The modified immuno-chromogenic screen is sensitive, specific and has led to the isolation of mutants E(M) over-secreting the α-sec-EAP reporter protein by a minimum of 50 fold higher levels than that exported by non-mutagenized E(P) parental strains. Unselected proteins were also over-secreted.

## Introduction

The *Pichia* genera of yeast has been extensively used as an expression platform [1-4]. The *Pichia* system has been applied to producing viral, bacterial, yeast, rodent, human and plant proteins with more than 500 proteins (up to 270 Kd size) being produced in *P.pastoris* alone [2,5]. This biological platform has several advantages such as support of high-level production of native eukaryotic proteins of diverse functions [2] that are functional as they are appropriately glycosylated qualitatively and quantitatively unlike proteins produced in *E. coli* and *S. cerevisiae*[2-4]. However, all proteins are neither expressed equally well nor are they appropriately modified in *P.pastoris* and may require complex induction routines. The methylotrophic yeasts including *P. pastoris,* share a specific peroxisomal methanol utilization pathway which can be drawn on for optimizing production of any given protein [2-4]. In *P. pastoris* recombinant protein coding sequences are inserted by one-step replacement of genomic coding sequences at the Alcohol Oxidase *AOX1* locus with expression being regulated by the plasmid borne copy of the *AOX1* Promoter (*Paox1*). Recombinant protein production is based on exhaustion of glycerol as a carbon source thus maximizing cell density followed by induction of the *AOX1* promoter with methanol. In *P. pastoris* the *Paox1* promoter is repressed by glucose. Thus optimizing levels of protein production in *P.pastoris* are dependent on tedious preliminary studies requiring and selection from a wide array of vectors, host strains, construction, induction and purification protocols which are highly empirical and often protein specific [2,3,6,7]. Other complications have also been detected in the expression of proteins in *P. pastoris.* A solution is to draw on the power of well established protocols for mutagenesis in yeast genetics and to design a generic strain optimized for producing most if not all heterologous proteins using a generic recombinant protein as a marker for export and *AOX1* regulatory sequences regulating expression.

The secretory pathway consists of highly regulated steps including bi-directional Golgi- Endoplasmic Reticulum exchanges of cargo proteins, selection of cargo proteins into ER derived vesicles, coating of cargo proteins with specific sorting signals and exclusion of resident proteins from vesicles, peroxisomal biogenesis among others [8-18]. The export of the yeast α factor pheromone is an integral part of mating type switching mechanisms at the *MAT* locus and its secretion signal is also highly regulated [19-21]. Secretion of the α factor is dependent on a sequence of events including transport between the ER and Golgi that in turn is dependent on distinct interactions with Emp24p and Erv25p and cleavage of the secretory signal from the prepro-α factor at specific sites by specific proteases [10,19-21].

An alternative approach to laborious systematic optimization is the screening of mutants Retaining genetic lesions that are distributed in most of the above steps regulating secretion. Several distinct mutagenic screens for over-secretors have now been applied in the yeast species *Saccharomyces cerevisiae*, *Schizosacharomyces pombe* and *Pichia pastoris*. Of these the most comprehensive screens are available in *S. cerevisiae* and facilitated by the genome wide deletion arrays which (a) coupled with Flow Cytometry designed to gate cells in which a fluorescent cell surface ligand [22,23] extended the library size limits screened for over-secretors to 10^8^ cells/hour and mutants over-secreting by as little as 3 fold could be isolated, (b) a GFP-tagged plasma membrane protein expressed in a deletion library allowed the visual detection of mutants regulating, appropriate lipid modifications, delivery of the marker protein to the cell surface, the actin cytoskeleton and known membrane traffic regulators [24], (c) a genome-wide approach coupled screening for mis-sorting of proteins from the vacuole to the secretory pathway by visual inspection of halos of levels of secreted carboxypeptidase Y marker protein detected by immunoblotting of colonies [25], (d) the yeast secretion trap screen (YST) involved ligating any eukaryotic cDNA to a secreted invertase reporter gene, transforming the mutant invertase host strains and screening for complementation [10,26]. Other screens in *S. cerevisiae* included (e) Two hybrid screens [26], (f) chemical-genetic screens to identify small molecule inhibitors of secretion of a toxin [27] and (g) standard chemical mutagenesis followed by screening for stabilization of ERAD substrates [28].

In the absence of genome wide deletion libraries in *P. pastoris* alternative approaches were required for identifying and selecting over-secretors. These included conventional approaches of chemical mutagenesis combined with a positive selection for Peroxisome Biogenesis-Defective mutants - a putative secretory pathway organelle - by non-toxic intermediates in alternative methanol and formaldehyde utilization pathways [15,16,29]. Recently, a more novel vector based chromogenic screen for high level secretion of β-lactamase confirmed a correspondingly high level of co-expressed GFP encoded by linked sequences integrated at the *AOX1* locus [30].

We have taken a more conventional approach. Our goal was to isolate and characterize *P. pastoris* strains that are *generically biased* towards up-regulation of the secretion of *any* given heterologous recombinant protein, as long as it contains an appropriate signal peptide and is otherwise secretion-competent. We have applied a chemical mutagenesis screen for detecting genes in the *P. pastoris* genome that are presumptive homologues of the above genes regulating protein export in, *S.cerevisiae*, *S. pombe* and mammals. It is based on chemical mutagenesis of *P. pastoris* strains secreting a fusion protein consisting of the secretory signal of the *S.cerevisiae* α factor and the relatively robust *E.coli* Alkaline Phosphatase (EAP) as reporter which should allow for the convenient and direct scoring of protein export by Alkaline Phosphatase (AP) enzyme activity. Furthermore Human Placental Alkaline Phosphatase had been produced from culture supernatants of *P.pastoris* as a fusion protein with the endogenous secretion signal *PHO1/KEX2* encoded secretory processing signals [31-33].

The *PHO1/KEX2* encoded processing sequences of *P. pastoris* required for cleavage of α-*MAT* signal and export sequences and EAP Open Reading Frame (ORF) coding sequences were included in the construct in anticipation of efficient secretion of α-sec-EAP [34,35]. This reporter protein was designed to report *trans* acting mutations elevating export. After saturation mutagenesis of the *P. pastoris* genome with standard methods [36] seeding on selective and inductive media, secreted α-sec-EAP protein retained on nitrocellulose colony lifts required detection by an immuno-chromogenic variation of published methods [25,37-43]. The immuno-chromogenic screen was (1) specific for α-sec-EAP, (2) application of a common secondary antibody coupled to Calf Intestine AP for detecting both HSA and α-sec-EAP did not contribute background to HSA which at 10–216 fold higher levels is stained almost instantaneously, (3) with no detectable background (3 – 4 logs below full induction), (4) was highly sensitive detecting early secretion and 1.3 fold-1.6 fold differences in over-secretion, (5) responsive to levels of secretion directly related to levels of methanol inducer and (6) displayed high fidelity concordance in transfers of α-sec-EAP/HSA secreting colonies to inductive/screening, selective and rich media used for screens.

In a pilot study, 16,000 colonies were screened and 73 candidate clones were isolated; a number of the most intensely-staining colonies were chosen for further characterization. Enhanced secretion of α-sec-EAP was confirmed in three of the four clonally-purified isolates by several independent methods. These included inducible expression of α-sec-EAP detected by Gel Code Blue staining of total secreted proteins on PAGE-SDS gels, western blot detection of α-sec-EAP, and direct staining for α-sec-EAP activity with NBT/BCIP substrate on native gels current results [34,35]. Quantification of protein band intensities showed that the levels of secreted α-sec-EAP in the two characterized isolates were at least 50 fold higher than the levels secreted by the non-mutagenized parental strain. Densitometric comparisons with the known HSA standard in supernatants of H strains confirmed that the conservative estimates of levels of the secreted α-sec-EAP in the supernatants of the mutants ranged from 1.6 fold to more than 50 fold above the supernatant of the E(P) and the background protein detected in h-V control strain supernatants. This level of α-sec-EAP from E(M) was in the range of HSA at early time points. Trivial explanations for elevated α-sec-EAP activity such as consequences of bacterial contamination, structural and regulatory sequence changes, rearrangements and amplification of coding and flanking sequences of α-sec-EAP integrants at the *AOX1* locus have been excluded [34,35].

## Materials and methods

### Growth media and conditions

Media were prepared as per directions (Invitrogen Life Technologies Manual Version F) and with reagents supplied in the Multi-Copy *Pichia* Expression Kit (Invitrogen Life Technologies Catalog Number K1750-01). Minimal selective media included Minimal Dextrose medium (MD), Minimal Dextrose Agar (MDA), Minimal Methanol Medium (MM) and Minimal Methanol Agar (MMA). Enriched maintenance media included Yeast Peptone Dextrose (YPD), Buffered Minimal Glycerol-complex Medium (BMGY), Buffered Minimal Glycerol-complex Medium Agar (BMGYA). Enriched inductive media included Buffered Minimal Methanol-complex Medium (BMMY) Buffered Minimal Methanol-complex Agar (BMMYA). In both inductive media (MMA and BMMYA) concentrations of methanol titrated for maximal secretion of two exported proteins were established at 0.2% - 0.3% methanol and were routinely used in formulations of media. Inductive methanol media (MMA and BMMYA) for replica plate screening for over-secretor mutants were overlain with reinforced Nitrocellulose (NC) 82.5 mm discs (BioRad, catalog # 170–3202) and either termed MMA-NC or BMMYA-NC. The nitrocellulose filters were not sterilized as colonies were serially re-streaked in these screening protocols and internal controls excluded contaminants. Liquid cultures of cells were either grown in 50 ml centrifuge tubes or in flasks with shaking at 250 rpm and temperatures of 30°C or on plates in warm rooms at 30°C.

### Expression plasmids, antibodies and strains serving as controls for secretion of proteins –construction of the *GS115 pPIC9phoA* , E(P) parental reporter strain

The two integrative expression plasmids (pPIC9 and pPIC9K) used for constructing strains were capable of shuttling between *E.coli* and *P.pastoris* and insertion into the *AOX1* locus of *P.pastoris* by one-step transplacement, (Invitrogen Life Technologies Catalog Number K1750-01). α-sec-EAP secreting isolates) were either constructed or supplied as controls in the Multi-Copy *Pichia* Expression Kit (Invitrogen Life Technologies Catalog Number K1750-01), designated as, h (host), h-V (host-Vector), H (HSAsec), β-G (β-galactosidase) expressor, E(P) (α-sec-EAP secretor, Parental), α-sec-EAP over-secretor mutants, E(M9), E(M32) and E(M44), the non-mutagenized inactive PCR mutant EAP E(D2) and E(F2) their genotypes and phenotypes are summarized (Table 1).

**Table 1:** **Genotypes and phenotypes of *P.pastoris, *Isolates and Strains used**

***P. P.pastoris***		***P.pastoris *strain/**	**Colony**	**Genotype *his/met***	**Exported protein phenotype Or**	
	**Host strain -**	**Isolate- colony derived**	**Designations**		**Methanol utilization**	**Quantified**
	**Source**				**(Mut**^**S **^**or Mut**^**+**^**)**	**(see Figures**3**,**4**,**5**)**
1	GS115	host	h	*his4*, *Mut*^*+*^	His^-^, Mut^+^	UP1, UP3
2	GS115	vector-Host	v-H	*aox1::pPIC9K, HIS4, Mut*^*S*^	His^+^, Mut^s^	UP1, UP3
3	GS115	β-Galactosidase	β-G or (β-Gal)	*his4::pPIC9K-lacZ, HIS4, Mut*^*+*^	His^+^, Mut^+^ β-gal	-
4	GS115	Human Serun Albumin	H or (HSAsec)	*aox1::pPIC9K-HSA, HIS4, Mut*^*S*^	His^+^, Mut^s^, HSA	HSA, UP1, UP3
5	GS115	*E. coli* Alk. Phos.(Parental)	E(P)	*aox1::pPIC9-phoA (A3#27), HIS4, Mut*^*S*^	His^+^, Mut^s^, phoA^+^	α-sec-EAP, UP1, UP3
6	GS115	*E. coli* Alk. Phos.(PCR-Mut.)	E(D2)	*aox1::pPIC9-phoA (D2,TrlStop), HIS4, Mut*^*S*^	His^+^, Mut^s^, phoA^-^	-
7	GS115	*E. coli* Alk. Phos.(PCR-Mut.)	E(F2)	*aox1::pPIC9-phoA (F2,TrlStop), HIS4, Mut*^*S*^	His^+^, Mut^s^, phoA^-^	-
8	GS115	*E. coli* Alk Phos.(Mutant)	E(M9)-A	*aox1::pPIC9-phoA (A3#27), HIS4, Mut*^*S*^	His^+^, Mut^s^, phoA^+^	α-sec-EAP, UP1, UP3
9	GS115	*E. coli* Alk. Phos.(Mutant)	E(M9)-B	*aox1::pPIC9-phoA (A3#27), HIS4, Mut*^*S*^	His^+^, Mut^s^, phoA^+^	α-sec-EAP, UP1, UP3
10	GS115	*E. coli* Alk. Phos.(Mutant)	E(M32)-A	*aox1::pPIC9-phoA (A3#27), HIS4, Mut*^*S*^	His^+^, Mut^s^, phoA^+^	α-sec-EAP, UP1, UP3
11	GS115	*E. coli* Alk. Phos.(Mutant)	E(M32)-B	*aox1::pPIC9-phoA (A3#27), HIS4, Mut*^*S*^	His^+^, Mut^s^, phoA^+^	α-sec-EAP, UP1, UP3
12	GS115	*E. coli* Alk. Phos.(Mutant)	E(M32)-C	*aox1::pPIC9-phoA (A3#27), HIS4, Mut*^*S*^	His^+^, Mut^s^, phoA^+^	α-sec-EAP, UP1, UP3
13	GS115	*E. coli* Alk. Phos.(Mutant)	E(M32)-D	*aox1::pPIC9-phoA (A3#27), HIS4, Mut*^*S*^	His^+^, Mut^s^, phoA^+^	α-sec-EAP, UP1, UP3
14	GS115	*E. coli* Alk. Phos.(Mutant)	E(M44)-A	*aox1::pPIC9-phoA (A3#27), HIS4, Mut*^*S*^	His^+^, Mut^s^, phoA^+^	α-sec-EAP, UP1, UP3
15	GS115	*E. coli* Alk. Phos.(Mutant)	E(M44)-B	*aox1::pPIC9-phoA (A3#27), HIS4, Mut*^*S*^	His^+^, Mut^s^, phoA^+^	α-sec-EAP, UP1, UP3

Detection of secreted α-sec-EAP on NC filter immobilized membranes required correction for high levels of background endogenous phosphatase activity. Strains serving as negative controls for secretion correction were h *,* h-V and β-G which expresses (positive control for intracellular expression) but does not secrete (a negative control for export) β-galactosidase. Strain H was the positive control for secretion, titration of methanol induction, efficiency of colony replication and standard against which maximal levels of over-secretion were measured (1–2 gms/L of Human Serum Albumin). The Methanol utilization (Mut) phenotypes Mut^+^ of the β-G strain and the Mut^S^ of the H strain also served as negative and positive controls in screens for integrants at the *AOX1* locus required for optimal expression of heterologous proteins driven by the vector borne *Paox1* promoter. The E(P) wild type GS115 strain of *P.pastoris* inducible for *E.coli* strain K12 Alkaline Phosphatase (EAP) production and encoded by *E. coli* K12 *phoA* coding sequences (GenBank (gi 49175990) was constructed as detailed [34,35]. Briefly, the forward primer was designed to include the sequence interval between Xho I to SnaB I of the vector cloning site (1190–1214 bp) encoding the *PHO1/KEX2* processing sequence required for cleavage and efficient export. Genomic copies of *aox1*::pPIC9*phoA* and flanking sequences from three separate clones were sequenced and showed that two (D2 and F2) contained translational stops in all three reading frames. The third clone E(P) retained a complete Open Reading Frame for Mat α secretion signal-EAP and its regulatory sequences. These isolates were preliminarily designated as *aox1*::pPIC9*phoA* isolates E(D2), E(F2) and E(P).

Two primary (1°) and one common secondary (2°) antibodies were used for all the immuno-chromogenic stains and Western Blot Analyses. They are: Primary (1°) Rabbit-anti-EAP-IgG (Biodesign, catalog # K59413R) or primary (1°) anti-EAP in the text. Primary (1°) Anti-Albumin, Human (Rabbit), (Calbiochem catalog # 126584) or primary (1°) anti-HSA in the text. Secondary (2°) antibody Goat-anti-Rabbit-IgG-Alkaline Phosphatase Conjugate (Calf Intestinal Alkaline Phosphatase, Calbiochem cat # DC06L) or secondary (2°) anti-Rb-IgG-AP in the text.

### Establishment of sequence fidelity, copy numbers and site specific integration at the *AOX1* locus by *aox1::*pPIC9-PhoA integrants with Genomic Sequence, Long Template Polymerase Chain Reaction (LTPCR) and Southern Blot Analyses of genomic DNAs

#### Genomic sequence analyses, primer design and restriction maps

Nucleotide sequence organization of the *aox1*::pPIC9PhoA integrants at the *AOX1* locus including 2kb upstream of translational start and 2kb downstream of translational stop signals of the *AOX1* locus (proprietary sequence information Invitrogen) were assembled. Either commercially available (Multi-Copy *Pichia* Expression Kit (Invitrogen Life Technologies Catalog number K1750-01) or custom designed primers were used for amplification and sequencing by commercial services (Genewiz) and are described in detail elsewhere [34,35,44]. Nucleotide sequences were analyzed by BLAST2 sequences (NCBI) and JavaScript DNA Translator 1.1 programs. Restriction endonuclease maps of the composite *aox1*::pPIC9PhoA locus, were constructed with the Base Cutter Program (New England BioLabs) and where possible re-confirmed with published maps [1], (Invitrogen Catalog number K1750-01). Genomic DNA for cycle sequencing was extracted from mutant isolates and wild type strains by a DNA extraction method adapted for screening yeast clones by Polymerase Chain Reaction and sequenced as published [34,35,44].

Genomic DNAs were prepared by a modification of published methods for analyses of copy numbers and site specificity of integrants at the *AOX1* locus by Long Template Polymerase Chain Reactions (LTPCR) and Southern Blots and are described in detail elsewhere ([1,34,35,44,45]; Invitrogen Manual Version F).

Genomic templates and Primer combinations used for LTPCR analyses yielded full length amplicons from, the composite *aox1*::pPIC9-PhoA sequence, the 5^/^ flanks of *AOX1* and sequences encoding α-factor-EAP, the 3^/^ flanks of *AOX1* and from sequences encoding α-factor-EAP respectively. The methods and the sequences of the primers binding to the respective positions and genomic sequencing are discussed in detail elsewhere [34,35,42] and (Invitrogen Catalog number K1750-01). The above genomic DNAs were subjected to Southern blot analyses. Genomic DNAs from negative control host GS115 and host-vector (GS115: *aox1*::pPIC9K) strains and wild type and mutant strains were digested with a panel of restriction endonucleases selected from the *aox1*::pPIC9PhoA locus analyzed by the base cutter program of New England BioLabs (NEB). These enzymes yielded fragments of predicted sizes based on the composite sequence from the flanks, the coding sequences and joints of *aox1*::pPIC9-PhoA integrants with 5^/^ and 3^/^ flanks of the *AOX1* locus. Digested genomic DNAs were resolved by electrophoresis and transferred in duplicate to membranes by capillary methods as described [1,34,35,45]. Two sets of hybridization probes were generated by digesting pPIC9PhoA with enzymes to release the PhoA (EAP) coding sequences from the vector sequences. Both the insert and vector sequences were purified by agarose gel electrophoresis and separately hybridized to duplicate membranes, washed and autoradiographed as described in detail elsewhere [1,34,35,45].

#### Mutagenesis of the E(P) strain for over-secretor isolates

Standard protocols for mutagenesis of the yeast species *S.cerevisiae* were adapted for application to *P.pastoris*[35,36,46]. Cell densities, volumes and times of treatment with Ethylmethane Sulfonate (EMS) treated were titrated for maximal killing (95%) without irreversible loss of viability. These results were used for the protocol for generation of over-secretors. A single colony of E(P) was inoculated into 50 mls of BMGY medium, grown over approximately 40 hours washed twice in 0.1 M NaP0_4_ (pH7.0) buffer and resuspended to a final density of approximately 10^9^ cells / ml based on viable counts. A fixed volume of these cells (1.7 mls) was treated with 50 μL of EMS for 180 minutes. The EMS was inactivated (quenched) by withdrawing 100 μL of the cells into 4 ml of sterile 5% Na_2_S_2_0_3_ (Sodium Thiosulfate) solution. Viable counts established approximately 95% killing. These mutagenized cells (approximately 16000 colonies) were seeded on BMGYA at densities of approximately 400 colonies/100 mm plate screened for over-secretors by the method below. This initial screen yielded 73 over-secretor candidate colonies as evaluated by the immuno-chromogenic stain.

### Chromogenic and immuno-chromogenic stains for detecting proteins secreted by *P.pastoris* colonies on Nitrocellulose (NC) membranes

#### Chromogenic stains

As reactions between Alkaline Phosphatase and it’s substrate NBT/BCIP (Pierce catalog #34042) yields a readily visualized precipitate initial assays were based on direct demonstration of the enzymatic activity of EAP secreted by E(P), but not by negative control h, h-V, H and β-G control strains grided on plates of BMGYA-NC and the membrane was lifted onto BMMYA Petri dishes. After induction for 16 hours at 30°C the membranes were stained with NBT/BCIP for 5’ to 15’ and the staining terminated with washes in distilled water followed by Alkaline Phosphatase Stop Solution (Sigma Catalog # A-5852).

#### Immuno-chromogenic stains

Raising the specificity of staining for EAP activity required modifications including to published methods outlined in the abstract. The protocol is a modification of published methods [25,37-39,41-43]. For an initial standardization, replicate membranes with known test, positive and negative control grids of colonies grown on BMMYA plates with a range of concentrations of methanol were stained with antibody. Strains, H, α-sec-EAP non-expressor E(D2), E(F2) and secretor E(P) and negative control h-V, β-G and h were patched in replicate sets onto the top and bottom rows of the top and bottom halves of a BMGYA plate. This plate was sequentially replicated onto a series of BMMYA-NC plates supplemented with (0%, 0.01%, 0.025%, 0.05%, 0.075%, 0.1%, 0.2%, 0.3%, 0.5%, 1.0% methanol), MDA plates and BMGYA plates. Upper and lower halves of nitrocellulose membrane from each plate were respectively washed as described below and immuno-chromogenically stained by reaction with the antibodies for HSA with primary (1°) anti-HSA/ secondary (2°) anti-Rb-IgG-AP and for EAP with primary (1°) anti-EAP/secondary (2°) anti-Rb-IgG-AP followed by reaction with NBT/BCIP substrate which would also serve as substrate for immobilized endogenous phosphatases in simultaneous direct chromogenic staining as control for specificity. Colonies were washed off in 5 mls of TST buffer per membrane per petri dish for 5’-10’ at ~50 rpm and RT, (TST buffer 150 mM NaCl, 10 mM Tris (pH7.5), 0.1% Tween 20). Washed membranes were pre-adsorbed in 3–4 mls of 5% Milk TST buffer (TST buffer 150 mM NaCl,10 mM Tris (pH7.5), 0.1% Tween 20, 5% Milk (Carnation) per dish for 30’on a rotary shaker at RT or at 4°C. Membranes were lifted and halves (EAP specific stain - lower and HSA specific stain - upper) of the replicate series were reacted with the respective primary 1° antibodies (1°)anti- EAP (Rabbit α *E.coli* Alkaline Phosphatase, Biodesign cat #K59413R) and secondary (1°) anti-HSA (Rabbit α Human Serum Albumin Calbiochem Catalog # 126584) diluted to 1:5000 in 5% Milk-TST. This dilution was applied at 3 mls per membrane per Petri dish at RT for 45’-60’ while rotating on a shaker. The antibody dilution was poured off each membrane half bound with the respective primary (1°) antibody. Each membrane half was rinsed with TST buffer (3–4 mls of) and the buffer discarded. This washing step was repeated twice and washing continued on a rotary shaker for 5 minutes. The TST buffer was drained. The common secondary (2°) antibody binding to both primary (1°) antibodies, secondary (2°) ant-Rb-IgG-AP (Goat α Rabbit IgG–AP (Gt α Rabbit IgG covalently linked to Calf Intestinal Alkaline Phosphatase, Calbiochem cat #DC06L). The common secondary (2°) antibody binding to both primary (1°) antibodies was also applied at a dilution of 1:5000 in Milk TST buffer and 3 mls applied per membrane per dish at RT for 45’-60’ while rotating on a rotary shaker. The secondary (2°) antibody was discarded and the washing steps repeated in TST buffer as before. Each membrane half was stained with 3 mls NBT/BCIP one step staining mix (Pierce catalog #34042) per membrane on a rotary shaker for 5’-10’ at RT. The precipitated stain was visible within as little as 30” and overstaining avoided as this would mask distinctions between mutants secreting variable levels of protein proportional to staining in the subsequent screen for mutants. The substrate was washed off and the membranes rinsed several times with distilled water. Continued AP activity was retarded by adding Alkaline Phosphatase Stop Solution (Sigma Catalog # A-5852) or Terminator at 1:80 dilution. These changes restored the detection of specific proteins secreted by positive control strains without their detection from negative control strain presumably due to the suppression of background staining by endogenous phosphatases.

### Fidelity of replicates and concordance of colonies on inductive, selective and rich media as detected by the immuno-chromogenic assay

H cultures (400 colony forming units/88 mm plate) were seeded on BMGYA plates, incubated at 30°C for 48 hours and sequentially replicated onto BMMYA-NC, MDA and BMGYA plates with an oriented and sterile velvet (Bel Art Scienceware Replicating Velvets cat #37848-0001) with a replicating tool (Bel Art Scienceware Replicating Tool cat #37848-0000) and incubated for 24 hours at 30°C. The nitrocellulose filter was lifted off the plate and immuno-chromogenically stained for exported HSA with primary (1°) anti-HSA/secondary (2°) anti-Rb-IgG-AP and NBT/BCIP substrate as above.

### Immuno-chromogenic screen for E(M) over-secretors from mutagenized E(P)

Mutagenized cells were seeded on BMGYA at densities of approximately 400 colonies/100 mm plate for a total of approximately 16000 colonies and incubated at 30°C for 48–60 hours or until colonies were sufficiently grown for detection of secreted protein without the over-growth that would complicate isolating stained colonies. Each BMGYA plate was replicated onto an oriented and sterile velvet. The velvet was sequentially replicated onto labeled 88 mm Nitrocellulose membranes overlain on BMMYA plates and then onto BMGYA plates. Colonies were induced for 14–20 hours at 30°C. Membranes were lifted and colonies were immuno-chromogenically stained for immobilized α-sec-EAP with primary (1°) anti-EAP/secondary (2°)-anti-Rb-IgG-AP followed by reaction with NBT/BCIP substrate as above. Each membrane was stained with 3 mls NBT/BCIP one step staining mix without Levulinic acid (Pierce catalog #34042) per membrane on a rotary shaker for 5’-10’ at RT. The precipitated stain was visible within 30” and overstaining avoided as this would mask distinctions between mutants secreting variable levels of protein proportional to staining. The substrate was washed off and the membranes rinsed several times with distilled water. Continued AP activity was retarded by adding Alkaline Phosphatase Stop Solution (Sigma Catalog # A-5852, (also referred to as Alkaline Phosphatase terminator) at 1:80 dilution. Candidate over-secretor colonies staining above background staining colonies from the 1° screen were immediately picked and grided onto BMMYA-NC, MDA and BMGYA plates with the various GS115 strains included in the grids as positive and negative controls for secretion. Candidate colonies which were re-confirmed as over-secretors from this secondary (2°) screen were re-streaked onto BMMYA-NC and BMGYA stained and re-confirmed as over-secretors in tertiary (3°) and quartenary (4°) screens. Colonies were prioritized on the basis of staining intensities and homogeneity of staining within re-streaked sub-colonies and classified on the basis of plate number, position on the grid. This initial screen yielded 73 over-secretor candidate colonies.

### Confirmation of over-secretion of proteins into culture supernatants by candidate E(M) mutants

Methods for protein production were modified from standard protocols [2,3,41,47,48]. Isolated candidate colonies growing on MDA were inoculated into 6 mls of MD medium in sterile 50 ml centrifuge tubes and grown to saturation for 36 hours at 250 rpm and at 30°C. Cells were concentrated in GS-3 rotors in Sorvall RC-5B centrifuges at 1.6 K × g, 4°C for 10’ and re-suspended in 4 mls of BMGYM (supplemented with 15% glycerol). An aliquot of 2 mls of each re-suspension was frozen at – 80°C and the remaining aliquot was raised to 50 mls with standard BMGYM in 250 ml sterile culture flasks. Similarly grown aliquots of E(P), H positive control cells and h-V negative control cells were inoculated into 50 mls of standard BMGYM in 250 ml sterile culture flasks. Cultures in flasks with loosened lids were grown at 250 rpm and 30°C for 20 hours, the absorbance at A_600_ determined and the cultures re-adjusted to equivalent cell densities. Cultures were grown for another 30 hours or to A_600_ of approximately equivalent absorbance of 10 –14 units. Cells were concentrated in GS3 rotors and RC-5B centrifuges at 1000 g for 10’ and re-suspended to equivalent cell densities of 4 × 10 ^9^ cells/ml in 5 ml final volumes of BMMY inductive medium and incubated as before in sterile 50 ml centrifuge tubes Aliquots (500 μL) of induced cultures were drawn at 0, 42, 69 and170 and their optical cell densities determined. With the exception of the 0 hour time point the remaining culture media were re-supplemented with 500 μL of 5% methanol. Induced cell cultures were centrifuged at 16000 × g for 5 minutes in pre-weighed Eppendorf tubes, the supernatants collected in sterile Eppendorf tubes on ice and the pellets weighed. One aliquot of the supernatant was used to determine total protein concentration by Bradford analyses (BioRad catalog # 500–0006). Aliquots were used for Western Blot analyses and total protein analyses by Gel Code Blue (Pierce catalog # 24590) staining of proteins resolved by SDS Electrophoresis as described below. Levels of specifically induced (α-sec-EAP and HSA) and non-specifically induced Unselected Proteins (UP1 and UP3) secreted protein bands were determined with ImageJ (NIH) Densitometric software. All protein secretion quantification was normalized to either internal standards of known units of purified EAP enzyme or a specified protein and graphically plotted versus time with Excel software. Growth rates, cell densities, cell mass and total protein concentrations were monitored as an index of culture conditions during the growth and inductive phases.

### Immuno-chromogenic and chromogenic stain based confirmation of secretion of α-sec-EAP and secreted Unselected Proteins (UP) secreted in supernatants of E(P) and E(M) isolates by Western Blot and Gel Code Blue Staining analyses

Laemmli Loading Buffer (6 μL) was added to aliquots of each supernatant (20 μL) per time point, Pre-Stained Broad Range Molecular Weight Markers (BioRad catalog # 161–0318) and 0.08 units of purified *E.coli* strain C75 Alkaline Phosphatase (Takara Catalog # 2120A) in loading buffer making up a set of samples and qualitative and quantitative standards. Each set was electrophoresed through denaturing Tris Glycine 4%-20% gradient polyacrylamide gels (Invitrogen Novex Catalog # EC60261BOX) and the gels were stained with Gel Code Blue (Pierce catalog #24590). Stained gels were scanned and the standards and both specifically and non-specifically induced protein bands densitometrically quantified with Image J programs (NIH). Laemmli Loading Buffer (50 mM Tris HCl (pH 6.8) 100 mM DTT, 2% SDS, 0.1% bromophenol blue and 10% glycerol) was added to aliquots of, each supernatants (9 μL) per time point, Pre-Stained Broad Range Molecular Weight Markers (BioRad catalog # 161–318 and 0.03 units of purified *E.coli* strain C75 Alkaline Phosphatase (Takara Catalog # 2120A). Each set comprising of samples and standards was electrophoresed through standard denaturing polyacrylamide gels (15% polyacrylamide , acrylamide : bisacrylamide ratio of 29:1 , 5% SDS, 4% stacking and 15% resolving layers) with Tris-Glycine-SDS running buffer (25 mM tris, 250 mM Glycine (BioRad electrophoresis grade) (pH 8.3) and 0.1% SDS). Electrophoresed proteins were electro-blotted to buffer soaked nitrocellulose membranes (BioRad cat # 162–0115) between Whatman 3M paper sandwiches after pre-equilibration with transfer buffer (48 mM Tris base, 39 mM Glycine, 20% Methanol) under semi-dry conditions. Proteins were transfered at 250 mA for 40 minutes in a C.B.S. Scientifics semi dry blotter (model # EBU-4000). After transfer, membranes were rinsed in TST buffer and pre adsorbed in 5% Milk-TST and bound with the 1° antibody diluted 1:5000 in 5% Milk-TST buffer washed and stained with the secondary (2°) antibody binding to both primary (1°) antibodies i.e. Goat α Rabbit IgG –AP ( Gt α Rabbit IgG-Calf Intestinal Alkaline Phosphatase, Calbiochem catalog number DC06L) which was also applied at a dilution of 1:5000 in 5% Milk-TST buffer. After initial experiments showed that neither primary (1°) antibody to EAP (Rabbit α *E.coli* Alkaline Phosphatase, Biodesign catalog number K59413R) nor (Mouse Monoclonal Alkaline Phosphatase Anti-Alkaline Phosphatase Clone AP1B9, Sigma catalog number A2806) cross-reacted with the primary (1°) antibody to HSA (Rabbit α Human Serum Albumin, Calbiochem Catalog # 126584), primary (1°) antibodies to both proteins were simultaneously bound to filter immobilized proteins. After binding for 1 hour at RT on a rotary shaker the 1° antibody in 5% Milk-TST buffer was drained and the membrane washed with TST buffer on rotary shakers for 10 minutes. Washing was repeated 2 times -3 times. Proteins immobilized on the membrane were then bound for 45 minutes to 1 hour at RT on a rotary shaker with the secondary (2°) antibody in 5% Milk-TST buffer. The antibody was drained and the membrane washed with TST buffer on rotary shakers for 10 minutes as before. Washes were repeated 3 times. The washed membrane was drained of any residual buffer and stained with NBT/BCIP as before, with robust nutating for 5 minutes to 15 minutes depending on the level of EAP activity. The reaction was stopped as before and the image scanned and saved for quantification with Image J Densitometric programs (NIH).

## Results

### Exclusion of sequence and copy number and rearrangements in putative *aox1*::pPIC9*phoA* integrants in E(P) and E(M)

The α-sec-EAP reporter protein was designed to detect *trans* acting mutations causing export up regulation. To ensure efficient secretion of α-sec-EAP, *PHO1* processing sequences of *P. pastoris* required for efficient cleavage of α-*MAT* signal and export were introduced into the coding sequences in the *α-sec-EAP* construct. The predicted size of un-processed α-MAT-sec-EAP protein is 54.9 kD while the observed size of 49 kD is larger than the 45.9 kD size of EAP which was cloned without its 70 aa *E. coli* leader sequence. Therefore instead of *PHO1/LEX2* recognition sequences, amino acid sequences within the upstream α-MAT leader may facultatively serve for low efficiency cleavage necessary for secretion yielding α-sec-EAP.

Furthermore, either elevated catalytic activity of α-sec-EAP or up-regulation of *aox1*::pPIC9*phoA* integrants due to rearranged flanking, regulatory and coding sequences in E(P) and E(M) could result in elevated staining scored as over-secretion. Therefore we first excluded these possibilities with sequence, Southern Blot and LTPCR analysis of the integrants in genomic DNA [34,35]. Alignments of sequences of putative *aox1*::pPIC9*phoA* integrants in E(P), E(M32) and E(M44) with the sequences in the database confirmed that, with the exception of a single Dra I site, all sequences including the flanking, regulatory and coding sequences of these 3 isolates were indistinguishable from *aox1*::pPIC9*phoA* sequences deposited in the database. This sequence similarity extended between nucleotides -236 and +1858. This new Dra I site at nucleotide -206, was beyond all known regulatory elements of *Paox1*. Similarly, the presence of *aox1*::pPIC9*phoA* as single copy integrants in E(P), E(M32) and E(M44) was confirmed by Southern Blot Analysis of genomic DNAs probed with α-sec-EAP coding sequences [34,35]. Finally, LTPCR analysis with primers annealing to internal (cds) and flanking sites yielded predicted products, confirmed the presence of a single copy of *aox1*::pPIC9*phoA* integrants and their organizational identity in E(P), E(M32) and E(M44) genomic DNA at the *AOX1* locus [34,35]. These data are consistent with the *HIS4*, Mut^s^ phenotypes of E(P) and high level expression of integrants at *AOX1* as opposed to *HIS4*, Mut^+^ low level expression of integrants at *AOX2*.

### Rationale for mutagenesis

The secretory pathway consists of highly regulated steps including bi-directional Golgi-Endoplasmic Reticulum exchanges of cargo proteins, selection of cargo proteins into ER derived vesicles, coating of cargo proteins with specific sorting signals and exclusion of resident proteins from vesicles among others (7–18). The export of the yeast α factor pheromone is an integral part of mating type switching mechanisms at the *MAT* locus and is also highly regulated (19, 20 21). Secretion of the α factor is dependent on the cleavage of the secretory signal at specific sites by specific proteases (19–21).

We have designed a mutagenic screen for genes in the *P.pastoris* genome that are homologues of the above genes regulating protein export in, *S.cerevisiae*, *S. pombe* and mammals. It is based on mutagenizing *P.pastoris* strains secreting a fusion protein consisting of the secretory signal of the α factor and the relatively robust and stable activity *E.coli* Alkaline Phosphatase (EAP) as reporter. It was expected that this reporter would allow for the convenient and direct scoring of protein export based on its AP enzyme activity.

### Assays for detecting over-exported α-sec-EAP protein

#### The chromogenic stain is non-specific

**Figure 1:**
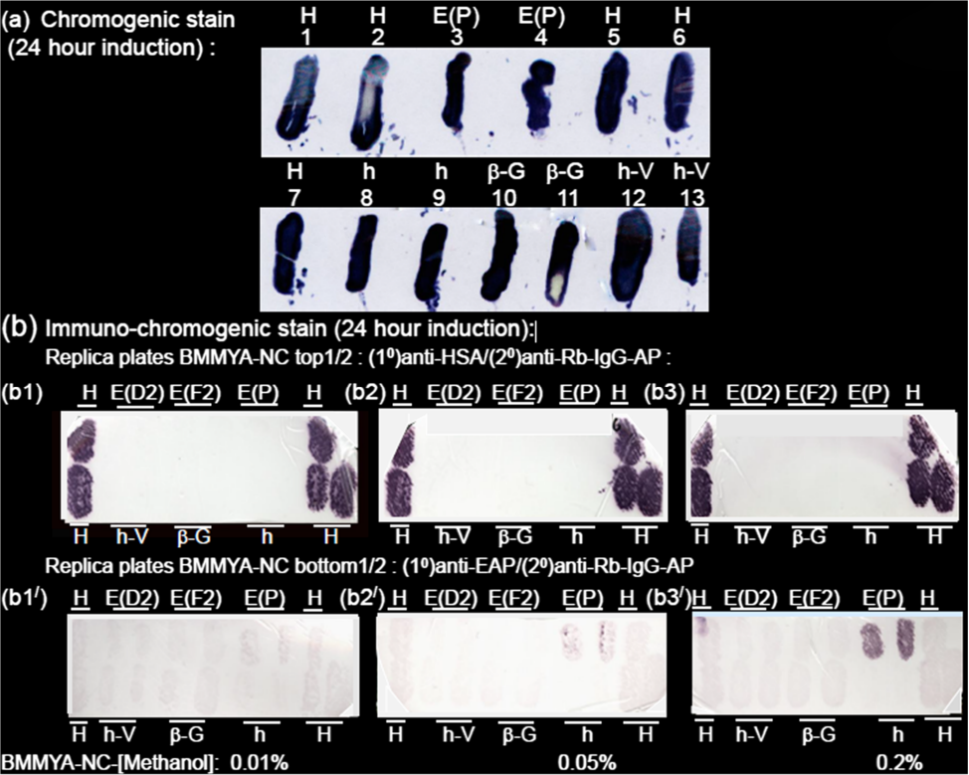
**Immuno-chromogenic but not chromogenic (direct) stains specifically detect exported proteins. **(Figure 1**a**) Chromogenic stain: Colonies derived from *P.pastoris *GS115 parental host strains (h) (patches 8, 9) and either strain *GS115-pPIC9K *Vector (h-V) (patches 12, 13) or exporting Human Serum Albumin, strain GS115-*HSAsec *(H) (patches 1, 2, 5, 6, 7) or parental strain *GS115-EAP(EA3#27) *(E(P) exporting α-sec-EAP (patches 3, 4) or the non-exporting strain *GS115-β-Gal *retaining intracellular β-galactosidase (β-G) (patches 10, 11) were patched onto BMGYA plates overlain with a nitrocellulose (NC) membrane. After 16 hours at 30^0^C the membrane was lifted onto a BMMYA plate and incubated for full induction at 30°C for an additional 24 hours. The NC membrane was then lifted onto Whatman 3MM Chromatography paper soaked with NBT/BCIP and monitored for differential staining of the strains. (Figure 1**b**) Strains H, α-sec-EAP non-expressor (translational stops in the sequence-E(D2), E(F2), α-sec-EAP expressor E(P), h-V, β-G and h were patched in replicate sets as labeled, onto the top and bottom rows of the top (Figure 1**b1**, **b2**, **b3**) and bottom (Figure **b1**’, **b2**’, **b3**’) halves of a BMGYA plate. This plate was sequentially replicated onto BMMYA plates overlain with nitrocellulose membranes (BMMYA-NC) and supplemented with (0%, 0.01%, 0.025%, 0.05%, 0.075%, 0.1%, 0.2%, 0.3%, 0.5%, 1.0% methanol), MDA plates and BMGYA plates. Representative methanol induction results of series of concentrations are shown in (1b1), (1b1^/^) at 0.01% (1b2), (1b2^/^) at 0.05% and (1b3), (1b3^/^) at 0.2%. Upper and lower halves of nitrocellulose membrane from each plate were respectively immuno-chromogenically stained by reaction with antibodies for HSA with primary/(1°)anti - HSA/(2°)anti-Rb-IgG-AP and for EAP with primary (1^0^)anti-EAP/secondary (2°)anti-Rb-IgG-AP followed by reaction with NBT/BCIP substrate which also serves as substrate for endogenous phosphatases in simultaneous direct chromogenic staining as control for specificity.

The ability to score for Alkaline Phosphatase (AP) activity in colonies by direct chromogenic staining with NBT/BCIP substrate was evaluated in preliminary experiments. The specificity of direct chromogenic staining for α-sec-EAP activity with the NBT/BCIP substrate was tested for staining between the non-expressor/non-secretor negative control vector or the host strain and either the positive control parental E(P) or H strains secreting α-sec-EAP and Human Serum Albumin (HSA) respectively. The parental putative positive control strain E(P) (Figure 1a, patches 3, 4) and negative control strains h (Figure 1a, patches 8, 9), h-V (Figure 1 a, patches 12, 13), H (Figure 1a, patches 1, 2, 5, 6 and 7) and β-G (Figure 1a, patches 10, 11) were chromogenically (directly) stained with NBT/BCIP as described in the materials and methods section. Serial exposures failed to show any differences between test, positive and negative controls which stained equivalently and intensely over a broad time period due to endogenous AP activities. As this endogenous AP activity will be shown to be essential for the mutagenic screen use of Levulinic acid inhibitor is excluded.

### The immuno-chromogenic stain is sensitive and specific for α-sec-EAP responsive to [methanol] inducer of *AOX1*

We introduced several modifications into the chromogenic stain to raise its sensitivity and specificity. Firstly, we converted into an immune antisera based assay that was directed against EAP activity using antisera. These were primary (1°) Rabbit-anti-EAP and secondary (2°) Goat anti-Rabbit-IgG-AP (Calf Intestinal Alkaline Phosphatase) antibodies. As the secondary 2° antibody was coupled to AP activity it yielded a 2^nd^ enzymatic source hydrolyzing the NBT/BCIP substrate and thus amplified the signal. Secondly, reduction of the cell/protein burden by washing off un-lysed colonies but not the exported and membrane immobilized protein elevated specificity. The level of membrane immobilized protein was directly proportional to the concentration of proteins exported into growth media (not shown, 34, 35). Thirdly, titration and reduction of the transcriptional inducer concentration of methanol to levels 10 fold lower than previous minimal levels of 0.5% in plate media allowed detection of exported α-sec-EAP and reduction of concentration of methanol in plates to 0.3%. Finally, restriction of staining to optimal concentration of antisera and staining period with Alkaline Phosphatase Terminator reduced accumulation of non-specific phosphatase stain.

**Figure 2:**
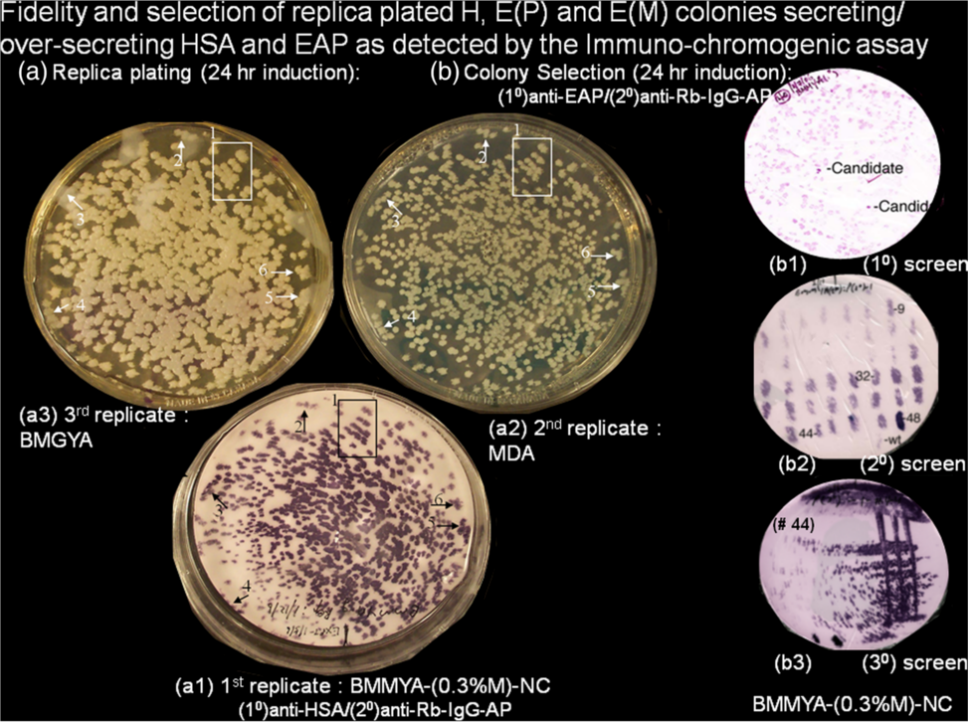
**Fidelity and selection of replica plated H, E(P) and E(M) colonies secreting/over-secreting HSA and EAP as detected by the immuno-chromogenic assay.** (Figure 2**a**) Concordance of colonies on inductive, selective and rich media. H (400 colonies/88 mm plate) were seeded on BMGYA plates (30°C, 48 hours), sequentially replicated onto (Figure 2a1) BMMYA-NC, (2a2) MDA and (2a3) BMGYA plates and incubated (30°C, 24 hours). (Figure 2**a**) The filter was lifted and immuno-chromogenically stained for exported HSA. Numbered white arrows (Figure 2a2, 2a3: 2–6) and open white boxes (Figure 2a2, 2a3: box 1) are matched to black arrows (Figure 2a1: 2–6) and box (Figure 2a1: box 1). (**b**) Selection of α-sec-EAP over-secretor mutants is independent of colony size and seeded strain on nitrocellulose membranes. Primary Screens (Figure 2b1) 400 colony forming units of mutagenized E(P) cells were seeded per 88 mm BMGYA plate and incubated (30°C, 24–36 hours). Colonies were sequentially replicated onto BMMYA-NC plates and BMGYA plates and incubated (30°C, 24 hours and 4°C, overnight). Membrane immobilized proteins were immuno-chromogenically stained for α-sec-EAP activity. Secondary Screens (Figure 2b2): Over-secretor candidates from the primary screen were identified by their intense immuno-chromogenic stain above host cell phosphatase as reference for α-sec-EAP activity. Seventy-three candidate colonies from the primary screen and non-mutagenized E(P) colonies as controls, streaked onto BMGYA plates placed on a 48 square grid and incubated (30°C, 20 hours). Colonies were replicated onto BMMYA-NC, MDA and BMGYA plates and incubated (30°C for 48, 24 and 24 hours, respectively). Immuno-chromogenicaly stained membranes, from BMMYA-NC plates confirmed elevated EAP staining by several of the candidate over-secretors relative to the P(E) strain. Three of these mutant E(M) candidates #9, #M32, #44 (Figure 2b2), were re-purified by streaking onto BMGYA plates, then sequentially replicated onto BMMYA-NC, MDA, and BMGYA followed by immuno-chromogenic staining. Purified single candidate colonies (2–4 replicates) (Figure 2b3), #9, #32 and #44) were inoculated for confirmation as over-secretors by Western Blot and Direct Staining with Gel Code Blue (GCB) staining of electrophoretically resolved proteins.

**Figure 3:**
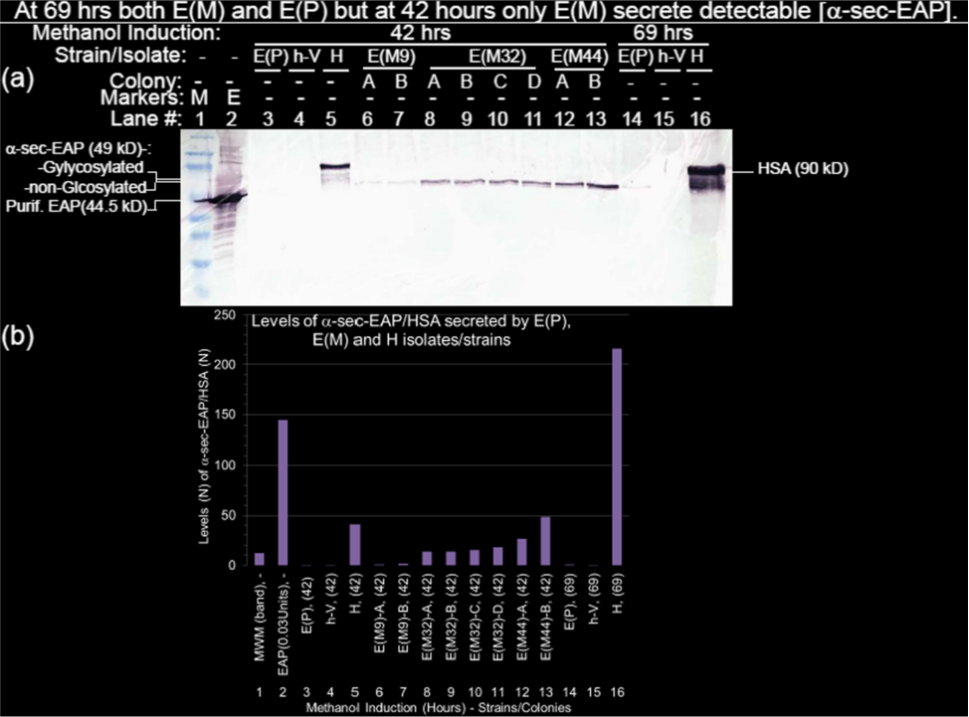
**At 69 hrs both E(M) and E(P) but at 42 hours only E(M) secrete detectable [α-sec-EAP]. **Culture supernatants of, E(P), h-V and H were harvested after methanol induction for 42 hrs (Figure 3**a**, lanes 3, 4 and 5) and 69 hours (Figure 3**a**, lanes 14, 15 and 16), 2–4 colonies per candidate E(M) mutants #9, #32 and #44 (from the re-streaked tertiary (3°) screen e.g. in Figure 2b3) after 42 hours of methanol induction (lanes 6 to 13) were electrophoresed, proteins transferred to NC membranes, sequentially annealed with primary (1°)anti-HSA/primary (1°)anti-EAP) and secondary (2°) anti-Rb-IgG-AP antibodies, washed, reacted with NBT/BCIP substrate and AP terminator as described in the materials and methods section. (Figure 3**b**). Levels of α-sec-EAP/HSA secreted by E(P), E(M) and H isolates/strains. The filter was scanned and saved as a TIFF file. Pixel densities of the proteins in the regions of interest (ROI) corresponding to, EAP (0.03 U) and MWM standards (Figure 3**b**, lanes 1, 2), α-sec-EAP (Figure 3**b**, lanes 3, 4, 6–15) and HSA (Figure 3b, lanes 5 and 16) in the Western Blot shown in Figure 3(**a**) were quantified with the NIH Image J Densitometric software. The respective pixel densities were normalized to E(P) at 69 Hrs (Figure 3b, lane 14) and plotted with Excel software. Based on this quantification the levels of secretion of α-sec-EAP confirmed the 3 different classes of E(M) mutants visually identified in the initial colony isolation as E(M9)-A, E(M9)-B (Figure 3**b**, lanes 6, 7), E(M32)-A, E(M32)-B, E(M32)-C, E(M32)-D (lanes 8–11), E(M44)-A and E(M44)-B (Figure 3**b**, lanes 12, 13).

The effects of these modifications are reflected in the results in Figure 1b. Colonies of strains/isolates H, E(D2), E(F2), E(P), h-V, β-G, and h were patched in duplicate or asymmetrically as indicated on the upper (Figure 1, b1, b2, and b3) and lower half (Figure 1, b1’, b2’ and b3’) grid of each plate representing the replicated series on the BMMYA-NC plates supplemented with increasing *AOX1* inducer concentration of methanol as identified in materials and methods. Representative results of inductions at 0.01%, 0.05% and 0.2% methanol (Figure 1, b1/b1’, b2/b2’ and b3/b3’) are presented. On the subsequent and sequential replication onto MDA and BMGYA controls grew as expected (not shown). Colonies on membranes from each plate were washed off and the grid of immobilized proteins on, the upper half reacted with the (1°)anti-HSA (Figure 1, b1, b2 and b3), on the lower half reacted with primary (1°)anti -EAP (Figure 1, b1’, b2’, and b3’), on both halves with the common secondary (2°) anti-Rb-IgG-AP and stained with NBT/BCIP as described in the materials and methods. Consistent with the growth of the full panel of replicated colonies on MDA and BMGYA the faint residual staining of immobilized endogenous phosphatases reveals the presence of the entire panel on the grid (Figure 1, b1’and b3’). This residual stain is not detected with anti-HSA, due to the much shorter staining period required by the significantly higher levels of secreted HSA (Figure 1, b1, b2 and b3). Although only low levels of background staining with primary (1°)anti-EAP are detected on E(P) patches at concentrations of methanol of 0.01% , specific staining for α-sec-EAP is detectable as speckling on the membrane at concentrations of methanol of 0.05% and clearly induced on the membrane at concentrations of methanol of 0.2% (Figure 1, E(P) b1’, b2’ and b3’). Immobilized proteins from the negative controls h, h-V, E(D2), E(F2), H and β-G were negative for staining at all 3 methanol concentrations, (Figure 1, b1’, b2’ and b3’, top row patches: H, E(D2), E(F2), H, bottom row patches: H, h-V, β-G, h and H). In contrast HSA is fully induced on the replicate membrane at concentrations of methanol of 0.01% and even 0% as detected with primary (1°)anti-HSA (Figures 1, b1, not shown). Further increases of concentrations of methanol of 0.05%, 0.2% and higher (upto 1%) do not increase the levels of HSA detected (Figure 1, b1, b2 and b3 and not shown). Similarly primary (1°) anti-HSA does not cross-react with immobilized proteins from the other negative control/secretor colonies (Figure 1, b1, b2 and b3, top row patches: E(D2), E(F2), E(P), bottom row patches: h-V, β-G, h). This despite secreted HSA levels likely being 10 fold 216 fold higher than the levels of α-sec-EAP on the same membranes (Figures 1, 2 and 3). Therefore the immuno-chromogenic stain for α-sec-EAP is not only specific with respect to non-detection of endogenous AP but also sensitive with respect to levels of α-sec-EAP being visually detected at 0.05% and 10 fold lower levels than the minimal 0.5% level specified. Furthermore it detects a level of secreted α-sec-EAP that is proportional to the level of *AOX1* transcriptional inducer concentrations of methanol which at 0.2% is lower than 0.5% recommended for BMMYA plates. We used 0.3% concentrations of methanol as it may make the assay more responsive for detecting small differences between the levels of α-sec-EAP secreted by E(P) and E(M) and immobilized on membranes.

### Growth and immuno-chromogenic staining of colonies sequentially replicated onto inductive /screening (BMMYA-NC), selective (MDA) and rich (BMGYA) media has good fidelity

Although NBT/BCIP is a cross-reacting substrate for several endogenous phosphatases which can be inhibited with Levulinic acid, we found it necessary to exploit this activity as the E(P) strain (to be mutagenized) only exports detectable levels of α-sec-EAP at 69 hours and not within the 36 hour period post-seeding required for effective screening. This requirement includes inclusion of a reference for rate and levels of export of α-sec-EAP, optimal growth, induction, colony staining, alignment, detection, resolution and purification. The endogenous cellular phosphatase activity effectively substitutes for these requirements. The results of applying these modifications are evident in Figures 2a and 2b.

The above standardization of the immuno-chromogenic assay, required first streaking colonies onto BMGYA master plates and then replicating the master plate onto test plates. The grids represented significantly higher cell density and mass per patch than the replicated colonies derived from the seeding of single cells during the mutagenic screen. This would yield correspondingly higher levels of secreted α-sec-EAP than that secreted by single colonies of highly variable size with corresponding variations in staining intensity complicating interpretations of size versus levels of secretion. As the H strain secretes high levels of HSA readily detectable in culture supernatants 20 hours post-induction, it was used to calibrate replication efficiency and baseline levels of staining intensity corresponding to colony size. BMGYA plates were seeded with approximately 400 H colonies, incubated at 30°C for 24–36 hours and then sequentially replicated onto screening/inductive (BMMYA-NC), selective (MDA) and rich (BMGYA) media (Figures 2, 2a1, 2a2 and 2a3). Replica plates were incubated for 24 hours at 30°C and the nitrocellulose membranes immuno-chromogenically washed and stained with primary (1°)anti-HSA/secondary anti-Rb-IgG-AP(2°) and NBT/BCIP substrate as before. Stained membrane immobilized HSA protein and replica colonies on MDA and BMGYA plates displayed a complete congruence in size, density and distribution (Figures 2, 2a1, 2a2 and 2a3, black and white squared outline 1 and arrows 2–6). Colonies of equivalent size stained with similar intensities and small colonies were as readily detectable as large colonies. Therefore the replication of colonies has fidelity and the immuno-chromogenic stain of the membrane can be reliably used under conditions of the mutagenic screen for colony variations in the levels of secreted α-sec-EAP protein.

### Mutagenesis of putative E(P) and screening for colonies over-secreting α-sec-EAP

The utility of exploiting the endogenous phosphatase activity for colony alignment and reference for rate and levels of over-secretion of α-sec-EAP was demonstrated by an example of the results of the mutagenic screen in Figure 2b. Approximately 16,000 colonies mutagenized with EMS by the standard protocols in the materials and methods section were seeded at densities of approximately 400 colonies per 88 mmm BMGYA plate. Plates were incubated until colonies were sufficiently grown for detection of secreted protein without the over-growth that would complicate isolating stained single colonies. Each BMGYA plate was sequentially replicated onto BMMYA-NC plates and then onto BMGYA plates which served as Master Plates for replication. Colonies were induced for 14–20 hours at 30°C, washed off and residual filter immobilized proteins including α-sec-EAP were immuno-chromogenically stained with primary (1°)anti-EAP/secondary (2°) anti-Rb-IgG-AP and NBT/BCIP as before. The progress of reactions was visually monitored and quenched to prevent masking of over-producers by over-staining of colonies either secreting basal parental levels of α-sec-EAP or the endogenous AP activity. Colonies that stained more intensely than equivalent sized colonies in a backdrop of uniformly staining colonies due to the endogenous phosphatase activity became candidates for over-secretors of α-sec-EAP (Figure 2b, 2b1, 1° candidates). This result represented the primary (1°) screen. Stained nitrocellulose membrane discs were wrapped in saran wrap, illuminated by light boxes and colonies from the corresponding BMGYA Master plates placed on the discs picked and sequentially patched onto inductive/screening (BMMYA-NC) and selective media (MDA) placed on oriented replication grids with appropriate positive and negative control colonies. The membranes were immuno-chromogenically stained with primary (1°) anti-EAP/secondary (2°)anti-Rb-IgG-AP and NBT/BCIP as before. Four of these colony grids stained significantly and variably more intensely than the parental E(P) positive control colony stained for endogenous phosphatase activity identifying candidate α-sec-EAP over-secretors (Figure 2b, 2b2, 2° candidates,colony patch numbers 9, 32, 44, 48 and wt). This result represented the secondary (2°) screen. We identified 73 candidate over-secretors of α-sec-EAP from this secondary (2°) screen. Colonies from the secondary (2°) screen were purified by re-streaking onto rich media and then sequentially replicated onto inductive/screening, selective and rich media as before. Nitrocellulose membranes were immuno-chromogenically re-stained for α-sec-EAP to confirm isolation of intensely staining colonies including colonies #9, #32, #44, and #48. (Figure 2b, 2b3, 3° candidates). This was the tertiary (3°) screen for candidate over-secretors of α-sec-EAP. A representative result, that for colony/patch # 44, is shown (Figure 2, 2b, 2b3). Supernatants from these colonies were tested for levels of secreted α-sec-EAP by Western Blot Analyses and direct staining with Gel Code Blue (GCB).

### Confirmation of over-secretion of α-sec-EAP by candidate 3° screen colonies by Western Blot analyses of culture supernatants

Confirmation of the candidates representing 4 levels of putative over-secretion in the tertiary (3°) screen involved measuring the levels of α-sec-EAP secreted into culture supernatants by Western Blot Analysis. Antibodies previously shown to be specific and sensitive were applied. Duplicate or quadruplicate colonies from some of the above 73 candidate over-secretor isolates were grown to saturation in MD broth, centrifuged and resuspended in BMGY-15% glycerol. Re-suspended cells were divided into two aliquots. One aliquot was frozen down and the second aliquot was used for confirmation of over-secretion of α-sec-EAP. The levels of total protein and α-sec-EAP in culture supernatants were quantified with Bradford and Western Blot analyses. Accumulation of HSA in supernatants of H relative to negative control h-V supernatant was clearly detected at the expected level of 1–2 gm/L. However, the background concentrations of exported unselected protein (UP) concentrations in the media apparently made detection of increases in α-sec-EAP relative to negative control h-V supernatant more difficult by standard Bradford Analysis for protein concentrations [34,35]. Therefore we proceeded to establish the presence of α-sec-EAP in the supernatants by Western Blot Analyses.

In initial experiments culture supernatants of 10 candidate over-secretor patch/colonies including numbers #9, #32, #44 and #48 were evaluated for the levels of α-sec-EAP by Western Blot analysis. Patch/colony #48 failed to reveal accumulation of α-sec-EAP in the supernatant and was excluded from subsequent analysis. These candidate over-secretors isolates were conditionally referred to as mutants E(M9), E(M32) and E(M44) hereon for convenience.

Molecular weight markers, chromatographically purified EAP (from *E.coli* strain C57) serving as quantitative, activity and size markers, supernatants from parental wild type, and H colonies serving as positive controls with h-V supernatants serving as negative controls were included in the tested panel. Supernatants were prepared from aliquots of cultures drawn at 0, 20, 42, 69, and 170 hours of induction with methanol. Electrophoresed gels were transferred to NC membranes and the same combination of antibodies that were used for immuno-chromogenic screening of colonies for HSA and α-sec-EAP were annealed to filter immobilized proteins. Western Blot analyses of the entire panel of supernatants induced for 42 hours (Figure 3a, lanes 3–13), E(P) and h-V and H for 69 hours (Figure 3a, lanes 14–16) are shown. Although the 90 kD HSA is clearly detected after 42 hours in strain H (Figure 3a, lane 5) α-sec-EAP is undetectable in E(P) at the Region of Interest (ROI) corresponding to 49 kD at 42 hours but becomes detectable at 69 hours (Figure 3a, lanes 3 and 14). This is the earliest time of detection. As expected, the ROI corresponding to α-sec-EAP in the negative control lanes of h-V at 42 and 69 hours remains undetectable (Figure 3a, 3b, lanes 4 and 15 Tables 2 and 3). HSA is detected as early as 20 hours and almost fully induced at 69 hours of induction of H with methanol (Figure 3a, Lane 16 and not shown). After 42 hours of induction secreted α-sec-EAP is clearly detected in all the mutant colonies E(M9)-A, E(M9)-B, E(M32)-A, E(M32)-B, E(M32)-C, E(M32)-D, E(M44)-A and E(M44)-B at the Region of Interest (ROI) corresponding to α-sec-EAP (49 kD) (Figure 3a, lanes 6–13). Furthermore visual inspection shows that the levels of the protein as reflected by the intensity of the bands corresponds to the staining intensity of the colonies in the secondary and tertiary screens that is E(M44 (-A,-B) > E(M32 (-A,-B,-C,-D) > E(M9(-A,-B) (Figure 3a, lanes 6–13). Chromatographically purified EAP from *E.coli* (strain C57) has a lower molecular weight than α-sec-EAP the *E. coli* (strain K12) *phoA* encoded EAP which may result from the retention of some amino acids from the α-MAT secretory signal. Furthermore the α-sec-EAP in mutant supernatants is present as a doublet that we have confirmed with Endoglycosidase H treatment results from the N-glycosylation of the lagging band. N-glycosylation of proteins is required for secretion in Eukaryotes but is not required in Prokaryotes [34,35].

**Table 2:** Western Blot (WB) Analysis of levels of α-sec-EAP & HSA secreted

**Lanes**	**Strain/colony/methanol(Hrs)**	**WB-EAP/α-sec-EAP/HSA (N)**
1	MWM (band), -	11.9692
2	EAP(0.03Units), -	144.25785
3	E(P), (42)	0.010629
4	h-V, (42)	0.010629
5	H, (42)	40.9967
6	E(M9)-A, (42)	0.98559
7	E(M9)-B, (42)	1.59943
8	E(M32)-A, (42)	13.46218
9	E(M32)-B, (42)	13.59085
10	E(M32)-C, (42)	14.9983
11	E(M32)-D, (42)	17.88467
12	E(M44)-A, (42)	26.1958
13	E(M44)-B, (42)	48.54267
14	E(P), (69)	1
15	h-V, (69)	0.010629
16	H, (69)	216.29118

**Table 3:** Mean levels of secreted proteins by WB & GCB staining in isolates/strains

**#**	**Isolate-Colony**	**(WB),HSA, α-sec- EAP, 42 Hrs EAP, (μ(σ)**	**(WB),HSA, α-sec- EAP, 69 Hrs EAP, (μ(σ)**	**(GCB), HSA, α-sec- EAP(m(s), 170 Hrs. EAP, (μ(σ)**	**(GCB), UP1, (μ(σ),170 Hrs**	**(GCB), UP3, (μ(σ),170 Hrs**
						
1	E(P)	0.01	-	1	1	1
2	h-V	0.01	-	1.76	0.55	1.15
3	H	40.99	-	279.11	2.04	3.2
4	E(M9), (m(s)	1.28(± 0.32)	-	1.8	0.67	1.62
5	E(M32), (m(s)	14.98(±1.78)	-	10.77(±1.31)	1.03(±0.79)	1.67(±0.16)
6	E(M44), (m(s)	37.37(±11.17)	-	57.09(±0.78)	3.79(±0.19)	1.16(±0.26)
7	E(P)	-	1	-	-	-
8	h-V	-	0.01	-	-	-
9	H	-	216.29	-	-	-

**Figure 4:**
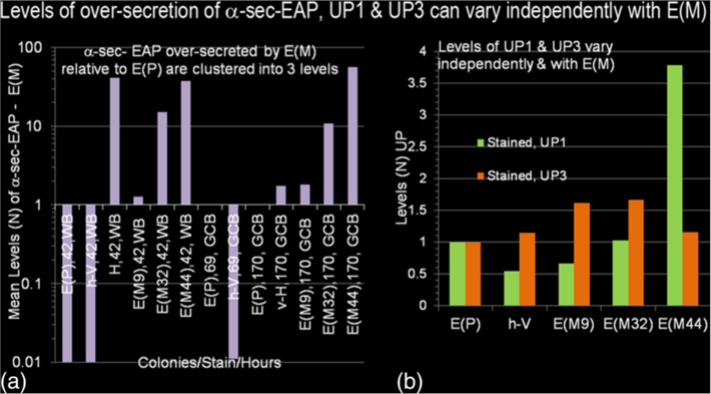
**Levels of over-secretion of **α**-sec-EAP, UP1 and UP3 can vary independently with E(M).** Values or mean values of ROIs corresponding to α-sec-EAP and HSA (42 and 69 hours inductions) from Western Blot Analysis normalized to E(P) 69 hours (Figure 3) and Gel Code Blue stained gels from 170 hour inductions normalized to UP3 from E(P) (Figure 5) were plotted as semi-log scales (Figure 4**a**, graphs WB and GCB). Plotted values were taken from tables 2, 3 and 4. However, to permit graphical resolution of low values, results of HSA secreted after 69 hours and 170 hours of induction were omitted from the plots. Mean values of UP1 and UP3 expressed as their ratios in E(P)/E(P), v-H/E(P) and all E(M)/E(P) were plotted (Figure 4**b**).

To confirm these visual differences in the levels of α-sec-EAP secreted by E(P), E(M9)-A, E(M9)-B, E(M32)-A, E(M32)-B, E(M32)-C, E(M32)-D, E(M44)-A, E(M44)-B, E(M44C), E(M44)-D and HSA by H at 42 hrs and/or 69 hours of induction detected in the Western Blots we quantified the above gel with Image J densitometry (NIH). The densitometric values were normalized to the value of E(P) at 69 hours of induction and graphically plotted with Excel programs. The ROI corresponding to α-sec-EAP in the negative control strain h-V (Figure 3a, 3b, lanes 4 and 15 Tables 2 and 3) and the parental strain E(P) (Figure 3a, 3b, lanes 3 and 14; Tables 2 and 3) define parental α-sec-EAP background or no activity at 42 hours while h-V also does not display any activity at 69 hours. The high specificity of the Western Analysis and the complete lack of background at the ROI of α-sec-EAP is dramatically illustrated by the logarithmic plot which shows >2 log lower pixels at E(P) (42 hours) and h-V (42 hours and 69 hours) (Figure 3a, lanes 3, 4 and 15 and Figure 4a Bars 1, 2 and 8).

Graphically, the strain E(P) at 69 hours, which is the first time the reporter is detected, from the parental strain, defines basal levels of α-sec-EAP and serves as the normalization standard (Figure 3b, lane 14). Visually the level of α-sec-EAP secreted by E(P) and E(M9) are similar, however when quantified the value for E(M9) at 42 hours, was higher than for E(P) at 69 hours (mean and maximal levels of 1.28(± 0.32) fold and 1.6 fold respectively) (Figure 3b, lanes 6, 7 and 14; Tables 2 and 3; Figure 4a, E(P) 69 hours). Although these are low levels of differences, the detection of over-secretion of α-sec-EAP by E(M9) is consistent with their independent detection above endogenous phosphatase activity of colonies in the primary (1°) mutagenic/immuno-chromogenic screens. Finally, when the ROI corresponding to secreted α-sec-EAP in h-V and E(P), E(M) and HSA secreted by H at 42 hours or E(P) and h-V at 69 hours and normalized to E(P) at 69 hours are plotted logarithmically the magnitude of the difference between these levels and the background at the ROI is 2–3 logs and clearly underestimated by linear plots (Figure 4a, graphs E(P) h-V, E(P) and h-V; Tables 2 and 3). As the high levels of expression of HSA at 69 hours (216 fold above E(P)) diminishes the resolution of these earlier time points it has been excluded from the plot (Tables 2 and 3). Mean values of immuno-chromogenically stained Western Blot quantification confirm the three classes of E(M) with the mean secreted levels of α-sec-EAP by E(M9), E(M32) and E(M44) being 1.3, 15 and 37.4 fold greater than that secreted by E(P) at 69 hours respectively (Table 2; Figure 3b, lanes 6–13; Figure 4a).

**Figure 5:**
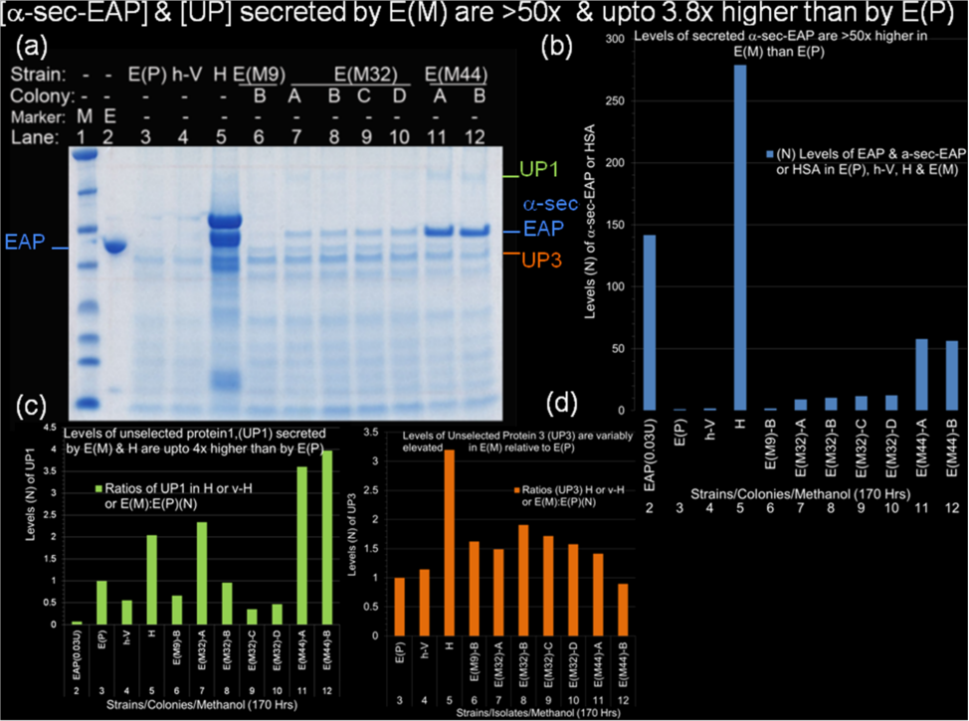
**[α-sec-EAP] and [unselected proteins,UP] secreted by E(M) are >50 and upto 4 fold higher than those secreted by E(P). **(Figure 5**a**) Incubation of the panel of cultures, shown in Figure 3 (with the exception of E(M9)-B, in lane 6) was for 170 hours with supplementation of methanol inducer at 24 hour intervals. Culture supernatants were harvested as determine in the materials and methods. Aliquots (20 μL) of cultures of E(P), h-V, H, E(M9)-B, EM32)-A,-B,-C,-D and E(M44)-A,-B were loaded in the order shown above the lanes (lanes 3–12) and co-electrophoresed with broad range markers (lane 1) and EAP purified from *E.coli *(0.08 units/lane, lanes2) through denaturing (4%-20%) polyacrylamide gradient gels with Laemmli electrophoresis buffer. The gel was stained with Gel Code Blue (GCB) scanned and saved as TIFF files. The respective ROIs of proteins corresponding to EAP, α-sec-EAP, unselected protein-1 (UP1) and unselected protein 3 (UP3) are identified and color coded on the left and right of the gel. The ROIs corresponding to the above 4 proteins were densitometrically scanned and normalized to UP3 in lane 3 and plotted as their respective ratios to E(P). Normalized ROIs corresponding to EAP, α-sec-EAP and HSA in E(P), v-H, H and E(M) were plotted with Excel programs (Figure 5**b**). Normalized ROIs corresponding to UP1 and UP3 were plotted as their ratios in EAP/E(P), E(P)/E(P), v-H/E(P), H/E(P) and all E(M)/E(P) (Figure 5**c**, 5**d**).

Strain H secretion of HSA, at 1–2 gms/L, is one of the maximal levels of known protein concentrations secreted by *P. pastoris*. HSA secretion at 42 and 69 hours is 40.9 and 216.3 fold higher than α-sec-EAP secreted by E(P) at 69 hours respectively (Figure 3a, 3b, Table 2 and 3). At least 1 mutant isolate E(M44)-B produced equivalent levels of α-sec-EAP to HSA at 42 hours at 48.54 and 40.99 respectively (Figure 3a, 3b lanes 5 and 13; Tables 2, 3). However, at 69 hours the level of HSA, was significantly higher than the level of α-sec-EAP secreted by E(M44)-B (Figures 3a, 3b, lanes 14, 16 and not shown; Table 3). In each of the three classes of mutants, the maximal levels of α-sec-EAP that were over-secreted by the colonies E(M9)-B, E(M32)-D and E(M44)-B were 1.6, 15 and 48.5 fold respectively higher than that secreted by E(P) at 69 hours (1 fold) to which they were normalized (Figure 4a, 4b, lanes 1–16, Table 3). Of interest the level of α-sec-EAP secreted by E(M44)-B is 1.9 fold higher than by E(M44)-A which is a duplicate isolate of the same colony. This difference is not detected by direct staining of aliquots of the same supernatants with Gel Code Blue staining (Figures 5a, 5b, lanes 11, 12, Figure 3a, lanes 12, 13, Tables 2, 3, 4). A more complete kinetic study of rates and extents of secretion of α-sec-EAP by these mutants has been completed and confirms these results [34,35] (Figure 3a, lanes 12, 13, Figure 4a, lanes 11, 12, Tables 3, 4).

**Table 4:** UP are variably over-secreted relative to α-sec-EAP in the panel of E(M)

**Lanes**	**Strain/colony/**	**GCB Levels of EAP & α-sec- EAP or HSA, in E(P),**	**Levels of UP1 in E(P),**	**Levels of UP3 in E(P),**
	**Methanol (70 Hrs)**	**h-V, H & E(M)**	**E(M), h-V, H,**	**E(M), h-V, H,**
		**(N)**	**(N)**	**(N)**
2	EAP(0.08U)/lead edge	141.7591	0.0757	0.0197
3	E(P)	1	1	1
4	h-V	1.7566	0.5539	1.0984
5	H	279.1085	2.0422	1.4002
6	E(M9)-B	1.7995	0.6655	2.0953
7	E(M32)-A	8.8916	2.3354	1.8787
8	E(M32)-B	10.2686	0.9618	2.3738
9	E(M32)-C	11.5889	0.3545	2.0224
10	E(M32)-D	12.3115	0.4713	1.9284
11	E(M44)-A	57.8638	3.606	1.7049
12	E(M44)-B	56.309	3.9762	1.0414

### Direct staining with Gel Code Blue confirms levels of over-secretion of α-sec-EAP and unselected proteins (UP) by E(M) isolates

Quantification of proteins by Western Blot analyses is qualified by several factors. These include variations in efficiency of transfer of proteins, variations in affinities of antisera and the retention of immuno-reactivity of denatured epitopes after electrophoresis and electroblot transfer. Similarly these factors affect ‘in gel’ enzyme activity stains [34,35]. This necessitates independent confirmation of the levels of α-sec-EAP over-secreted by E(M) by an approach without transfer and immune-reactive detection using methods such as direct chromogenic staining and quantification. In addition direct staining also allows the quantification of secretion of proteins that are not selected for over-expression (unselected proteins (UP) and do not cross-react with the antisera used for detecting α-sec-EAP in the immuno-chromogenic stain. Furthermore, as we have applied a generalized chemical mutagenesis screen which would generically affect secretion regulation, UP would also be expected to show variations in export. Therefore we quantified levels of 2 prominent secreted proteins (UP1 and UP3) in aliquots of culture supernatants by direct staining of aliquots of electrophoresed supernatants with Gel Code Blue (GCB). Incubation of the entire panel of strains and colonies analyzed by Western Blots was continued upto 273 hours with the withdrawal of aliquots and supplementation of growth media with methanol inducer at approximately 24 hour intervals [34,35]. Aliquots from the entire panel of culture supernatants (except E(M9)-A) from 170 hours of induction and co-electrophoresed with known units of chromatographically purified EAP as molecular mass standard, molecular weight standards through pre-cast gradient polyacrylamide gels and stained with GCB (Figure 5a, lanes 1–12, identified on left and right margins). The ROI corresponding to the EAP standard, α-sec-EAP, HSA, UP1 and UP3 were densitometrically quantified with Image J (NIH), normalized to UP3 in E(P) and plotted with Excel as a function of their ratios in E(P) i.e. protein in E(M):protein in E(P) or protein in H: protein in E(P) (Table 4, Figures 5a, 5b, 5c, 5d, lanes 2–12, color coded and identified on sides of gel). These results of direct staining with GCB were consistent with those of Western Blot analysis of the supernatants at 42 hours. GCB staining confirms that the levels of over-secretion of α-sec-EAP by E(M32) and E(M44) were 8.9-12.3 fold and 56.3 - 57.9 fold higher than secretion by E(P) (Table 4, Figures 5a, lanes 2–12, Figures 4a, 4b). However, although clearly elevated by 1.8 fold and visible as a protein band, the ROI corresponding to α-sec-EAP in E(P) and E(M9)-B also displays a co-migrating protein at an equivalent level in the non-mutagenized negative control h-V lane (Figure 5a, 5b, lanes 3, 4, 6; Figure 4 and Table 4). Therefore this is probably an alternative protein co-migrating with the ROI corresponding to α-sec-EAP. Therefore staining with GCB is neither sufficiently sensitive nor selective enough to detect the over-secretion of α-sec-EAP by E(M9) that was previously confirmed with antisera and Western Blot Analysis (Figures 3a, 3b). In contrast the mean levels of α-sec-EAP over-secreted at 170 hours by E(M32) and E(M44) were 10.77(±1.31) and 57.09(±0.78) respectively (Figure 5a, 5b, lanes 7–12; Figure 4 and Table 3). Thus the levels of α-sec-EAP secreted by E(M44) at 170 hours is only 2.5 fold below and thus in the range of the levels of 0.08 units of purified EAP standard corresponding to a level that is 141.8x higher than the corresponding ROI in E(P) (Figures 5a, 5b, lanes 2, 11 and 12, Table 4). In contrast, the level of HSA, secreted by H at 170 hours is 1.97x higher than the purified EAP standard, 279.1x higher than the levels of ROI of α-sec-EAP secreted by E(P) and 4.9 fold higher than the α-sec-EAP secreted by E(M44) (Figure 5a, 5b, lanes 2, 5, 11 and 12; Figure 4 lane omitted; Tables 3 and 4).

Saturation mutagenesis would be expected to yield multiple mutations/cell. Given the large numbers of target genes in the secretory pathway over-secretor isolates resulting from saturation mutagenesis would predictably include multiple independent mutations in the secretory pathway affecting the secretion of multiple proteins. Some of these mutations may specifically regulate the secretion of the α factor secretory signal thus resulting in the over-secretion of α-sec-EAP. To exclude the possibility that over-secretor mutations were specifically restricted to the α factor secretory signal we determined whether non-specific and unselected proteins (UP) in the screen for α-sec-EAP over-secretors specifically UP1 (~135 kD) and UP3 (~44 kD) were also over-secreted. UP1 and UP3 are detected in the supernatants of all the cultures. Visual inspection of the gel shows that both UP1 and UP3 were over-secreted or under-secreted by E(M) relative to E(P) and to extents that were low and variable (Figure 4a, lanes 3–12, color coded). Although, these levels of UP1 and UP3 were variable, even within colonies of a single E(M), they were not in convergent directions/levels between and within colony supernatants and therefore could not result from generalized degradation of protein (Figure 5a, lanes 3–12).

To confirm these small variations in their levels of secretion UP1 and UP3 proteins were quantified, normalized to UP3 secreted by E(P) and plotted as described above and in the Materials and Methods section (Figure 5a, 5c, 5d; UP1 (green), UP3 (orange); Figure 5b; Table 4). Relative to UP3 from E(P) maximal mean levels of over-expressed UP1 were secreted by mutant E(M44) at 3.79(±0.19) which was 3.7 fold higher than that secreted by E(M32) at 1.03(±0.79) (Figure 4a, 4c, 4d; Table 3, Figure 5b, Table 4). This difference for UP1 secreted by E(M44) is even further elevated to 6.4 fold if the exceptionally high level of secretion by 1 of 4 isolates E(M32)-A is excluded to yield a mean secretion level of 0.596 (±0.0.263) (Figure 5a, 5c, 5d; Table 3, Figure 4b, Table 4). It is clear that E(M44) secretes UP1 by a minimum of 3.8 fold higher level, which is even higher than UP1 levels secreted by H strains while the mutant E(M9)-B and non-mutagenized negative control strain h-V under-secrete UP1 by factors of 0.7 and 0.6 fold (Figure 5a, 5c, 5d; Table 3, Figure 4b, Table 4).

Therefore if there is normal variation in secretion of UP3 in the mutants it consistently trends toward a small over-secretion excepting in supernatants from one isolate E(M44)-B which shows no effect (Figure 5a, 5c, 5d; Table 3, Figure 4b, Table 4). The mean levels of over-secretion of UP3 by E(M32) and E(M44) at 1.67(±0.16) and 1.16(±0.26) respectively peaked at maximum levels of, 2.37 fold for E(M32)-B, 1.7 fold for E(M44)-A and 2.1 fold for E(M9)-B (Figure 5a, 5c, 5d; Table 3, Figure 4b, Table 4). However, the level of over-secretion of UP3 by E(M44)-A was 1.6 fold higher than by an isolate from the co-purified (3° colony) streak E(M44)-B. This disparity between the two E(M44) colonies was neither paralleled by the levels of secretion of α-sec-EAP nor of UP1 which were respectively equivalent between the isolates (Figure 5a, 5c, 5d; Table 3, Figure 4b, Table 4). In contrast to under-secretion of UP1 by the negative control strain h-V, levels of secretion of UP3 were equivalent to E(P), and were maximal at 3.2 fold in H strains (Figure 5a, 5c, 5d; Table 3, Figure 4b, Table 4). The mutant isolate E(M44B) that secreted maximal levels of α-sec-EAP and UP1 secreted minimal levels of UP3 in the entire array of cultures excluding the introduction of either systematized or random errors in the quantification of these small differences. The trends in the patterns of over-secretion and under-secretion are illustrated by the parallel plots of UP1 and UP3 secretion from the entire array of culture supernatants (Figure 5a, 5c, 5d; Table 3, Figure 4b, Table 4). A more comprehensive kinetic study of protein secretion over 273 hours of induction in all the control, parental and mutant colonies extended and reinforced these results [34,35].

## Discussion

To screen for over-secretor mutants we had constructed a reporter parental strain E(P) secreting α-sec-EAP. However, α-sec-EAP secreted by E(P) was only detectable at 69 hours which was beyond the 36 hour period useful for screening mutants, when colony growth and resolution were optimal for their isolation. The possible reasons for either the delay in secretion or the elevation of secretion to detectable levels of α-sec-EAP by E(P) are addressed in greater detail elsewhere [34,35]. Furthermore, to screen for over-secreting E(M) we modified published protocols in 2 important ways. Firstly, we exploited endogenous cellular phosphatase activity as a reference for rates and levels of over-secretion of α-sec-EAP by E(M) as well as for colony alignment. Direct chromogenic staining for α-sec-EAP activity was non-specific due to the cross-reaction of endogenous phosphatases with the NBT/BCIP substrate of α-sec-EAP. This endogenous activity could be suppressed with Levulinic acid. However, instead, we exploited it to refine the screen. We used the endogenous yeast cellular phosphatase activities as a reference for rates and levels of secretion of proteins also detected with the Alkaline Phosphatase based immuno-chromogenic stain. Well known endogenous cellular (*P. pastoris*) general phosphatase activities which cross-react with the substrate NBT/BCIP of Bacterial Alkaline Phosphatase constituted this reference. Although the same secondary antibody coupled to Calf Intestine AP is used to detect both proteins, this background is not detected at the significantly lower times (instantaneous to seconds) of staining required for detecting HSA which is exported at higher levels (10 to 216 fold) than α-sec-EAP. Secondly, merely by converting the chromogenic stain to an immuno-chromogenic stain we raised the levels of sensitivity and specificity. This was further elevated by supplementing and further amplifying the AP activity of the α-sec-EAP reporter protein with the Calf Intestinal AP cross-linked to the 2° antibody in the immune reaction based immuno-chromogenic stain. Thus two sources of AP hydrolyze/tautomerise the BCIP/NBTsubstrate, elevating the yield of the colorimetric product and amplifying sensitivity. In a further departure from published methods we titrated inducer levels, intensity of stain in relation to colony size and fidelity of replica plating. Raising the specificity of staining for α-sec-EAP activity required four additional modifications to published methods that were outlined in the abstract and the materials and methods sections [25,37-39]. These included cultivation on the membrane instead of colony/plaque lifts allowing secreted proteins to accumulate on the membrane [39]. Furthermore, unlike published protocols, the calibrated levels showed that despite a 10 fold reduction of minimal concentrations of methanol inducer of the *AOX1* transcription unit, exported α-sec-EAP protein was detectable by the immuno-chromogenic stain as speckles in patched colonies [25,37,38]. This titration of concentrations of methanol displayed a clear and direct visual relationship with the levels of α-sec-EAP secreted by patches. Furthermore, we confirmed by Western Blot Analysis, the detection of a mutant E(M9) over-secreting low levels (1.3 fold) of α-sec-EAP at 42 hours relative to the levels secreted by E(P) controls at 69 hours when the reporter became detectable. This level was sufficient for initial detection of α-sec-EAP over background endogenous cellular phosphatase activity by the independent primary (1°) screen thus validating the resolving capacity of the immuno-chromogenic screen. These three criteria tend to validate E(M9) as a low level over-secretor. Therefore the immuno-chromogenic screen is at least as sensitive as equivalent methods [25,37-43]. An extensive kinetic study over 273 hours quantitatively confirmed a >100 range range of expression of the three classes of mutants over the parental E(P) strain at 42 hours of induction. Finally, we demonstrated fidelity of replica plating and detection of secreted proteins independently of colony size (as opposed to patches) on immuno-chromogenically stained membranes, selective and rich media used for screens in contrast to most other methods [25,37-43]. These modifications in the chromogenic stain to yield an immuno-chromogenic screen of mutants was also specific for α-sec-EAP, with no detectable background (negative controls are three to four logs below full induction), high sensitivity of detection (1.3 -1.6 fold differences) in over-secretion at earlier time points and no cross-reaction with HSA secreted at 10–216 fold higher levels on the same membrane. Application of this chemical mutagenesis and immuno-chromogenic screen to 16,000 colonies in a pilot study, yielded 73 candidate over-secretor clones. We have ruled out trivial explanations such as *cis* acting sequence changes in the *aox1*::pPIC9*phoA* integrants in E(P) and E(M), contamination with bacterial proteins and endogenous phosphatase activity [34,35].

Enhanced secretion of α-sec-EAP reporter protein was confirmed in three of the four clonally-purified isolates by several independent methods. Supernatants from colonies of the fourth mutant isolate, E(M48), which was the most intensely stained colony in the secondary (2°) screen, failed to reveal detectable α-sec-EAP upto 273 hours of induction (not shown). We interpret the stain as resulting from cell lysis during washing yielding immobilized membrane debris retaining α-sec-EAP with the exceptionally high AP activity that was observed that is consistent with the robust nature of Bacterial Alkaline Phosphatases. This activity may not be exported and is therefore detected on the grid but not in the supernatant. Further work on the putative mutant E(M48) was suspended.

Quantification of α-sec-EAP confirmed three distinct levels of enhanced secretion of α-sec-EAP by the remaining three mutants as predicted by a general chemical mutagenesis screen. Confirmation of these over-secretion mutants included inducible expression and molecular weight of α-sec-EAP detected by Gel Code Blue staining of total secreted proteins on PAGE-SDS gels and Western blot detection of α-sec-EAP. In one variable result, although there was a 1.9 fold difference in the levels of α-sec-EAP secreted by duplicate isolates E(M44)-A and E(M44)-B as quantified by Western Blot analysis, equivalent levels of α-sec-EAP were detected when the same supernatants were directly stained with Gel Code Blue. We interpret this as a reduction in the antigenicity but not in mass due to possible denaturation and aberrant re-folding of α-sec-EAP masking epitopes from E(M44)-A but not from E(M44)-B during electrophoresis or transfer. Extended kinetic studies of α-sec-EAP secretion over 273 hours, N-glycosylation and direct in gel chromogenic staining for α-sec-EAP activity with NBT/BCIP substrate on native gels further confirmed both over-secretion and the catalytic function of the secreted protein [34,35].

Quantification and logarithmic plots of immuno-chromogenically stained Western Blots showed that maximal levels of α-sec-EAP were three to four logs higher than the background stain represented by E(P) and h-V at 42 hours of induction. GCB stained gels showed that the levels of α-sec-EAP secreted by two of the characterized isolates E(M44)-A and E(M44)-B were at least 50 fold higher than the levels secreted by the non-mutagenized parental strain. These levels increased substantially at later time points. Furthermore comparisons with the HSA secretion standard, known to be secreted at 1–2 gms/L peak levels, in supernatants of H strains confirmed that the conservative estimates of levels of the secreted α-sec-EAP in the supernatants of the E(M44) mutants were in the same range at 42 hours of induction. Trivial explanations for elevated α-sec-EAP activity such as consequences of sequence changes, rearrangements and amplification of coding and flanking sequences of α-sec-EAP integrants at the *AOX1* locus have been excluded [34,35]

A general de-regulation of protein export in E(M) mutants, would be expected to also affect proteins normally exported by *P.pastoris*. Two proteins normally exported by non-mutagenized parental E(P), negative control h-V and positive control H strains are the unselected proteins UP1 and UP3. We monitored variations in the secretion of UP1 and UP3 by the three classes of mutants relative to E(P), h-V and H. The same supernatants in which α-sec-EAP secretion was quantified also showed low level (1.5 -3.8 fold) but distinct over-secretion of UP1 and UP3 by E(M). However the trend towards over-secretion of maximal UP1(3.8 fold) by E(M44) was reversed to a low 2 fold level of under-secretion by all the other E(M9) and E(M32) mutants. The exception was the 2.4 fold over-secretion of UP1 by E(M32A). In contrast all the E(M) mutants over-secreted UP3 by 1.4-1.8 fold with the exception of E(M44B) which still over-secreted UP1 and α-sec-EAP maximally. Although these differences are small the trends of over-secretion and under-secretion of UP1, UP3 and α-sec-EAP are clearly visible (Figure 4a, 4b). Furthermore, although non-mutagenized E(P) and h-V also displayed small 2 fold variations in levels of secretion at 170 hours a more extended time course failed to sustain this minimal difference while retaining a kinetic profile [34,35]. Similarly secreted levels of UP1 and UP3 were significantly elevated in supernatants of non-mutagenized H strains. This may either reflect the elevated background tracking in the lane or the overburdened export functions of the Endoplasmic Reticulum-Golgi systems by the high levels of HSA (1–2 gms/L) secreted by H strains leading to elevated generalized secretion. This concern is not applicable to E(M44) as elevation of α-sec-EAP also elevates UP1 but decreases UP3 secretion.

Finally, we tested 50 ml inoculums of the E(P), E(M) test isolates, the negative control h-V, and positive control H (HSAsec) representing one of the highest known levels of exported protein at 1–2 gms/L) strains for secretion of total protein by BioRad Protein Quantification Assays over an extended time course (unpublished results, 34, 35). By normalization to total protein exported by h-V, representing background levels, total protein secreted by E(M) and H were consistent with those recorded in Table 4 showing that E(M44) over-secretes within 5x of the range of secretion of HSAsec strains [34,35]. Although the export of unstable proteins, unlike α-sec-EAP, and poorly secreted proteins (such as those lacking α-factor secretory domains e.g. UP) may not translate to scale there is no reason *a priori* to believe that these results will not extrapolate to large scale production of proteins by E(M44). This is only a short techniques note, but in subsequent papers we will elevate this 50 ml scale to 1L shaker and 5L fermenter cultures similarly to the production methods of human Cathepsin K (catK) from standard *P.pastoris* GS115 host strains that we and others have applied [47,48]. From the results of elevation of scale of production of catK and at least 13 mutants of this enzyme, in wild type *P.pastoris* GS115 host strains, as well as recombinant protein production from *P. pastoris* that is reported in the literature, α-sec-EAP and other recombinant proteins produced from E(M44), the levels of secretion observed at 50 ml scale should predict yields from mid scale and large scale cultures [47,48].

## Conclusion

We have taken the more conventional approach of chemical saturation mutagenesis and screening for over-secretion of the reporter protein α-sec-EAP by a modified immuno-chromogenic method. This result has yielded mutants which over-secrete the reporter protein to significant levels. The over-secretion was not restricted to the reporter protein but also included an unselected host protein. Therefore we have established the novelty and viability of the approach while identifying mutants which may be useful in over-producing any generic protein under these growth and regulatory conditions.

## Competing interests

Neither author has any known competing interests.

## Authors’ contributions

SA specified project goals, provided general outlines, reagents, materials and lab, assisted with data analysis and interpretation. FN planned, surveyed literature, designed immuno-chromogenic assay, was responsible for experimental design and execution, data analysis and interpretation and preparation of manuscript. Both FN & SA were in complete concordance with all the information in this and subsequent manuscript (in preparation) which have been reviewed, presented at the meetings listed in the references and are in preparation for a second manuscript (referenced here) since 2006. Both authors read and approved the final manuscript.
